# Supplementary figures and images for: CGIAR Barley Breeding Toolbox: A diversity panel to facilitate breeding and genomic research in the developing world
Source: Front Plant Sci. 2022 Nov 14;13:1034322. doi: 10.3389/fpls.2022.1034322 (PMC9702823; doi:10.3389/fpls.2022.1034322)

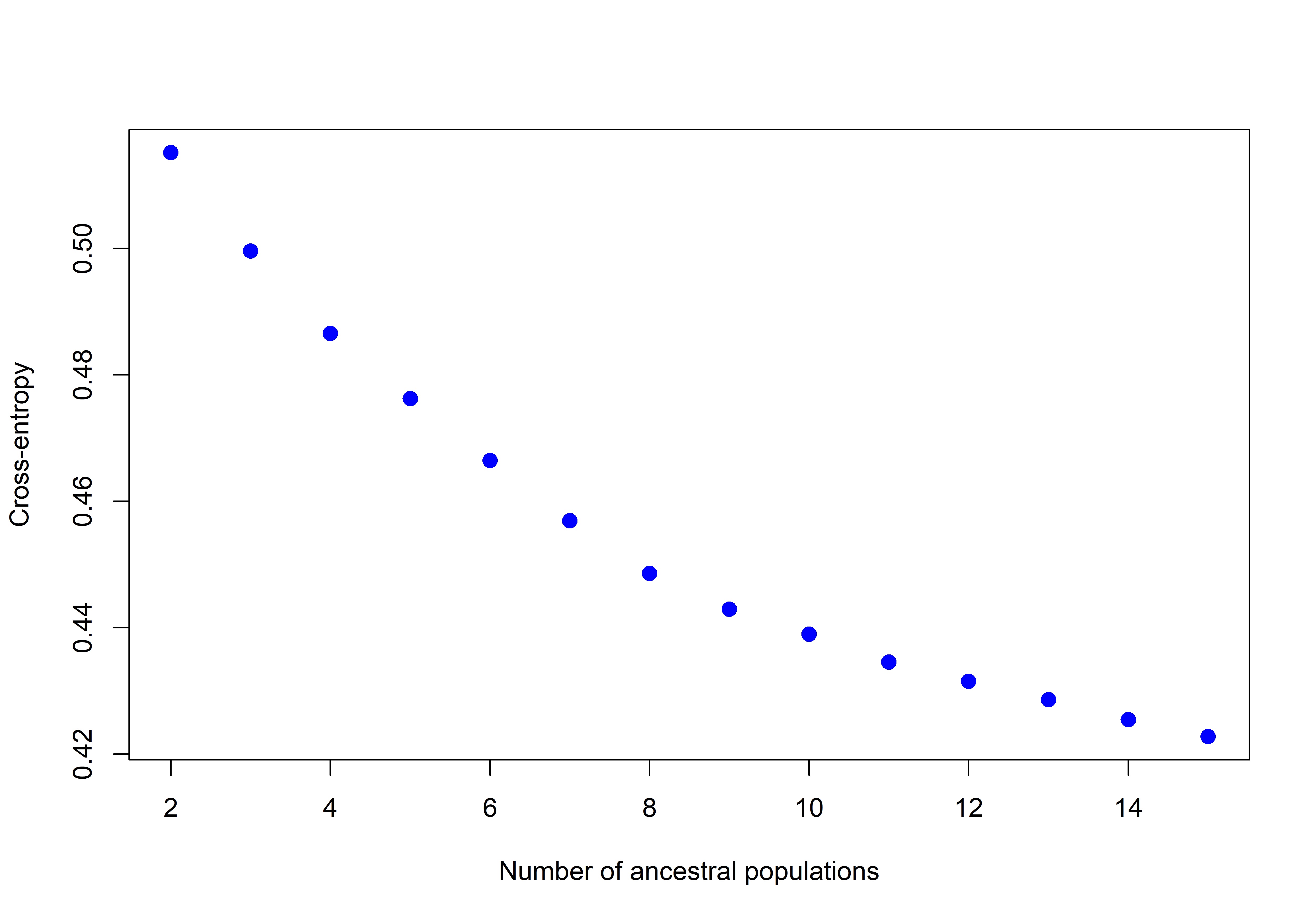

Supplement: Supplementary file 2 [file Image_1.jpeg]
